# Inhibition of Food Spoilage Fungi, *Botrytis cinerea* and *Rhizopus* sp., by Nanoparticles Loaded with *Baccharis dracunculifolia* Essential Oil and Nerolidol

**DOI:** 10.3390/foods13213403

**Published:** 2024-10-25

**Authors:** Aldrey Nathália Ribeiro Corrêa, Naiara Jacinta Clerici, Natália Oliveira de Paula, Adriano Brandelli

**Affiliations:** 1 Laboratory of Nanobiotechnology and Applied Microbiology, Institute of Food Science and Technology, Federal University of Rio Grande do Sul, Porto Alegre 91501-970, Brazil; aldrey.correa@ufrgs.br (A.N.R.C.); naiaraj.clerici@gmail.com (N.J.C.); nataliaodepaula@gmail.com (N.O.d.P.); 2 Center of Nanoscience and Nanotechnology, Federal University of Rio Grande do Sul, Porto Alegre 91501-970, Brazil

**Keywords:** antifungal activity, *Baccharis dracunculifolia*, *Botrytis cinerea*, nerolidol, nanoparticle

## Abstract

This study investigates the antifungal potential of encapsulated essential oil (EO) from *Baccharis dracunculifolia* and nerolidol (NE) within Pluronic^®^ F-127 nanoparticles (NPs). The EO, containing nerolidol, β-caryophyllene, and α-pinene as major bioactive compounds, exhibited superior antifungal activity compared to NE. The NP-EO formulations demonstrated high efficacy against *Botrytis cinerea*, with inhibition rates ranging from 29.73% to 87.60% and moderate efficacy against *Rhizopus* sp., with inhibition rates from 11.81% to 32.73%. In comparison, NP-NE showed lower antifungal activity. Both formulations effectively inhibited spore germination, with NP-EO showing greater inhibition compared to NP-NE. The encapsulation efficiency was significantly higher for NP-EO (80.1%) as compared to NP-NE (51.1%), attributed to the complex composition of EO facilitating better encapsulation and retention. Stability studies indicated that both NP formulations were stable at 25 °C for at least 15 days and exhibited changes in particle size and the formation of smaller particle populations at other temperatures (4 °C and 37 °C). Hemolytic activity was low across all NPs, suggesting their safety for food applications. The findings underscore the efficacy and applicability of EO-encapsulated NPs in extending food shelf life and maintaining product quality. The controlled and prolonged release of active compounds, coupled with their antifungal activity and safety, suggests that these NPs represent a promising and innovative approach for food preservation and active packaging development.

## 1. Introduction

Filamentous fungi are microorganisms of greatest concern for food safety as lower eukaryotic heterotrophic organisms that rely on external carbon sources [1]. These microorganisms are responsible for several plant diseases and cause the spoilage of fruits, vegetables, and grains, resulting in huge economic losses [2]. After spore germination, the fungi directly penetrate the plant epidermis through natural openings or wounds, as well as through specialized structures such as appressoria. They either attach to the fruit surface for penetration or use enzymes to degrade the plant cell walls [3]. Two of the recurrent filamentous fungi responsible for fruit spoilage are *Botrytis cinerea* and *Rhizopus* sp., which cause significant losses, particularly in strawberry, tomato, and grape cultivars [4]. There are few alternatives for controlling fungi in food due to either chemical residues or challenges in application. Therefore, it is essential to develop innovative solutions to reduce fungal contamination in fresh produce, substrates, and food products [4,5].

The natural antimicrobials currently receiving attention are the essential oils (EOs) [6,7]. EOs are derived from natural plant-based raw materials through various extraction processes, including mechanical (citrus fruits, orange, lemon, bergamot, lime, tangerine, and grapefruit), physical (aromatic plants, basil, and thyme), and chemical methods (fragile components of flowers, not separable by heat), as well as dry distillation (woods, barks, roots, or gums). Steam distillation is the most common extraction method, and it has been employed for obtaining several EOs, such as herbal leaves, mint, oregano, and tea tree [8]. EOs contain a complex mixture of natural polar and non-polar substances, volatile compounds, terpenes, including monoterpenes and sesquiterpenes, aromatic compounds (such as methoxy derivatives, aldehydes, alcohols, and phenols), and terpenoids [4,9].

**Figure 1:**
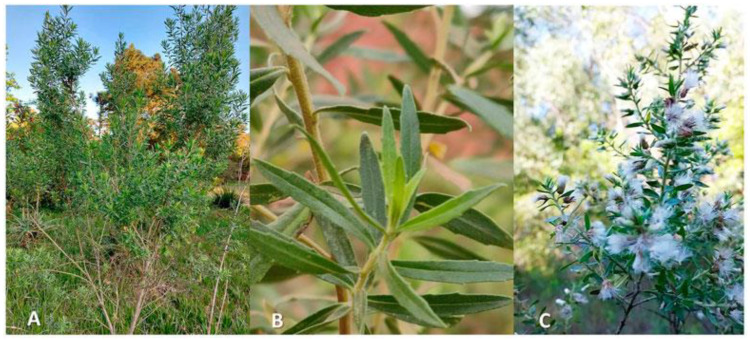
Adult specimen (**A**), leaves (**B**), and flowers (**C**) of *Baccharis dracunculifolia* DC. Reproduced from Gazin et al. doi: 10.3389/fphar.2022.1048688 [11], under the terms of the Creative Commons Attribution License (CC BY).

The plant *Baccharis dracunculifolia* (Figure 1) belongs to the Asteraceae family and the *Baccharis* genus, which includes approximately 500 species. It is widely distributed in South America, particularly in the southern region of Brazil, and in countries such as Argentina, Uruguay, Paraguay, and Bolivia [10,11]. This plant is culturally regarded as a source of natural compounds and is traditionally used in the treatment of various diseases [6,11]. Moreover, it has been demonstrated that its secondary metabolites possess antimicrobial, antioxidant, antiparasitic, anti-inflammatory, and antiviral activities [11,12,13,14,15]. The natural sesquiterpene alcohol nerolidol (3,7,11-trimethyl-1,6,10-dodecatrien-3-ol) is the main compound found in the EO of *B. dracunculifolia* and is permitted by the US Food and Drug Administration (FDA) as a safe flavoring agent in foods [12,16]. Nerolidol has a wide range of pharmacological and biological activities attributed to it, such as antioxidant, antibacterial, antibiofilm, insecticide, and antiparasitic properties, being of great interest in the field of agriculture and medicine [16].

There has been a growing demand from consumers for foods without synthetic additives and preservatives [17]. The food industry has significant interest in EOs and their constituents, as the use of these bioactive compounds as natural antimicrobial agents has been described in various studies [18,19,20]. However, the interaction of pure EO with the food matrix can cause undesirable organoleptic effects when added in quantities sufficient to provide an antimicrobial effect [21]. In this context, nanoencapsulation appears among the alternatives for delivering natural antimicrobials [22]. Therefore, applying EOs in the form of nanoparticles can mask the sensory attributes of the EOs and their components, enhancing apparent solubility and stability while reducing interaction with the food matrix. This approach has the potential to increase biological activity, bioaccessibility, and bioavailability [22,23].

Among the polymers used in nanotechnology, the triblock copolymer Pluronic^®^ F-127 appears as an interesting material in developing nanoparticles with antibacterial activity against foodborne bacteria [24,25], being effective in encapsulating geraniol to control enteric bacterial pathogens on spinach surfaces [26]. It is an inexpensive polymer with high biocompatibility and low toxicity, approved by the FDA for use in various pharmaceutical applications, including oral products [24,27]. However, its application for the delivery of natural compounds with activity against food spoilage fungi has been poorly explored [28,29]. Therefore, the aim of this study was to evaluate the inhibition of *B. cinerea* and *Rhizopus* sp. using Pluronic^®^ F-127 nanoparticles loaded with EO from *B. dracunculifolia* and its major compound, nerolidol (NE).

## 2. Materials and Methods

### 2.1. Chemicals

The *B. dracunculifolia* EO was obtained from Harmonia Natural (Canelinha, Brazil). Nerolidol (98%, mixture of *cis* and *trans*), Pluronic^®^ F-127 (powder, BioReagent, suitable for cell culture), 2,2-diphenyl-1-picrylhydrazyl (DPPH) and 2,2′-azino-bis-(3-ethylbenzothiazoline)-6-sulfonic acid (ABTS) radicals, and 6-hydroxy-2,5,7,8-tetramethylchroman-2-carboxylic acid (Trolox) were purchased from Sigma Aldrich (St. Louis, MO, USA). Tetrahydrofuran (THF) and Potato-Dextrose agar (PDA) were obtained from Merck (Darmstadt, Germany). Polysorbate 80 (Tween 80^®^) was acquired from Synth (Diadema, Brazil). Capric/caprylic triglyceride was provided by Delaware (Porto Alegre, Brazil). Ultrapure water was obtained using a Milli-Q purifier (Millipore, Burlington, MA, USA). All other reagents were of analytical grade, and ultrapure water was used to prepare all solutions.

### 2.2. Microorganisms

The filamentous fungi *Botrytis cinerea* and *Rhizopus* sp. were isolated from grape and tomato, respectively. The strains were obtained from the culture collection of the Laboratory of Toxicology (ICTA-UFRGS, Porto Alegre, Brazil). Fungal identification was performed as described elsewhere [30] based on macroscopic and microscopic morphological criteria and molecular analyses of the internal transcribed spacer (ITS) region and part of beta-tubulin gene. PDA plates were inoculated and incubated at 25 °C for 7 days or 15 days for mycelium and spore analyses, respectively [31]. The isolates were maintained on PDA agar plates under refrigeration (7 °C) and subcultured in a fresh plate prior to each experiment.

### 2.3. Characterization of the EO

The manufacturer provided the chemical characterization and quantification of the EO compounds. The analysis was performed through gas chromatography coupled with mass spectrometry using Agilent MSD5977B equipment (Agilent, Santa Clara, CA, USA). Compounds were separated using a DB-5MS capillary column (30 m × 0.25 mm × 0.25 µm). The injector and detector temperatures were set at 280 °C and 260 °C, respectively. The oven temperature gradient was as follows: an initial temperature of 60 °C (2 min) with a rate of 4 °C/min to 200 °C, and then a rate of 6 °C/min to 260 °C (10 min). The ionization source temperature was 280 °C, and the acquisition mode was scan. A 1.0 μL aliquot was injected using the split mode (split ratio, 1:20). Compound identification was based on the comparison of mass spectra of peaks with those in the NIST17.L library (NIST Chemistry WebBook), with the similarity degree of each identification presented in the results table. The relative percentage area of each peak was calculated based on the sum of the areas of all peaks eluted from the column and originating from the analyzed sample, including peaks identified as “unidentified compounds” due to similarity values below reliable identification thresholds.

### 2.4. Antifungal Activity of EO and NE

#### 2.4.1. Agar Contact Method

Antifungal activity was assessed using the direct contact method on agar [31] with modifications. The EO or NE was added to PDA (containing 0.05% Tween 80) at concentrations of 0, 1, 5, 10, 20, and 25 mg/mL. Then, 15 mL of the PDA solution was poured into sterile Petri dishes (90 mm in diameter). A mycelial disc (3 mm in diameter) was removed from 7-day-old cultures on PDA plates using a punch and placed in the center of each Petri dish. The Petri dishes were incubated for 7 days at 25 ± 2 °C, with measurements of mycelial growth taken at 72 h, 5 days, and 7 days in two perpendicular directions (diameter in mm). Comparison of the obtained dimensions with those of the controls allowed for the calculation of the percentage of inhibition (*IP* %) at the end of the incubation period, according to Equation (1):

(1) $$IP\;\left(\%\right)=\frac{\left(C-T\right)}{C}\times 100$$

where *C*: average colony diameter (mm) in the control; *T*: average colony diameter (mm) in the treatment.

#### 2.4.2. Exposure to Volatiles

The antifungal activity of the volatiles from the EO and NE was also evaluated as described elsewhere [32]. Volumes of 5, 10, 20, and 25 µL were applied to filter paper discs (16 mm in diameter), which were then fixed to the center of the inner lid of Petri dishes containing solidified PDA. The plates were sealed with Parafilm to ensure a controlled environment. The volatiles were released from the impregnated paper discs into the dish during an incubation period of 7 days at 25 ± 2 °C. The pathogen inoculation, incubation, and growth measurement procedures were the same as those described for the direct contact method.

### 2.5. Preparation of Nanoparticles

**Figure 2:**
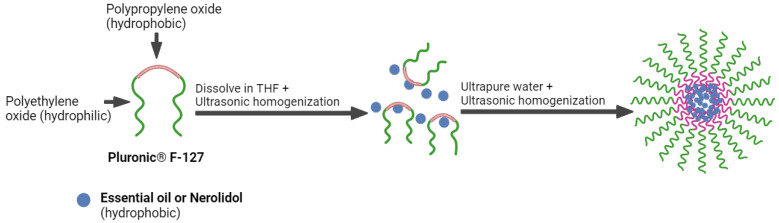
Schematic representation of the nanoparticle preparation process using Pluronic^®^ F-127, *Baccharis dracunculifolia* essential oil, or nerolidol.

The organic phase of the nanoparticle (NP) solution consisted of Pluronic diluted 1:1 (*w*/*w*) in EO or NE, followed by the addition of this solution to tetrahydrofuran (THF) at a ratio of 16% (*w*/*w*). The mixture was emulsified using a probe-type ultrasonic device (Unique OF S500, Unique, Americana, Brazil) operating at frequency 50 kHz, power 250 W, for two cycles of 5 min each with 2-min intervals [26]. The aqueous phase of the solution consisted of sterile ultrapure water. The organic phase was then added to the aqueous phase by the nanoprecipitation method at a 1:10 (*w*/*w*) ratio, followed by sonication for three additional cycles of 5 min with 2-min intervals to ensure complete dilution (Figure 2). The NP solution was subsequently placed under a hood for 20 h with moderate agitation to remove the THF. The NPs were collected, filtered through 0.22 μm membranes (Millipore, Billerica, MA, USA), and stored in sterilized glass containers at 25 °C before further use. In addition to the NPs containing EO (NP-EO) or NE (NP-NE), control formulations were prepared by replacing the EO/NE with caprylic/capric acid triglycerides (NP-B).

### 2.6. Characterization of the Nanoparticles

#### 2.6.1. Particle Size and Zeta Potential

To determine the size (average diameter), polydispersity index (PDI), and zeta potential (ζ), the NP suspensions were diluted in ultrapure water (1:10, *v*/*v*). The size was measured using Dynamic Light Scattering (DLS) with a goniometer (BI200SM, λ = 632.8 nm, 75 mW He-Ne laser, and BI 9000 correlator; Brookhaven, Nashua, NH, USA) at a fixed angle (*θ* = 90°). Data processing was performed using the NNLS. The cumulant method was used for PDI determination. The zeta potential was measured by electrophoretic mobility using a ZetaPALS Potential Analyzer (Brookhaven Instruments, Nashua, NH, USA) at 25 °C.

#### 2.6.2. Encapsulation Efficiency

Encapsulation efficiency (EE%) was determined in triplicate using the centrifugation filtration technique [33]. To determine the EE, it was necessary to identify the wavelength of maximum absorbance (λmax) for the EO and NE and to construct the standard curve. The λmax values were obtained by diluting the EO in methanol, followed by scanning with a UV/VIS spectrophotometer, where the maximum absorbance was detected at λ = 293 nm and λ = 262 nm for EO and NE, respectively. The standard curve was generated by diluting the EO or NE to concentrations of 0.0015, 0.003, 0.006, 0.012, and 0.025 mg/mL in methanol. The correlation coefficient (*r*^2^) was 0.9919 with the equation y = 2.9797x + 0.0011 for EO, and *r*^2^ was 0.9966 with the equation y = 3.78084x − 0.0054 for NE.

For the determination of EE, 500 μL of the NP suspension was added to centrifuge filters (Ultracel^®^-3k, Merck Millipore Ltd., Tullagreen, Ireland), followed by centrifugation at 10,000× *g* for 30 min at 4 °C. After centrifugation, the supernatant was diluted in methanol, and absorbance was measured using UV/VIS spectrophotometry at 293 nm and 262 nm for EO and NE, respectively, and quantified using the standard curve. The EE was calculated using the following Equation (2):

(2) $$EE\left(\%\right)=\frac{NP-S}{NP}\times 100$$

where NP is the total amount of EO or NE used in the preparation of the NPs (mg/mL), and S is the total amount of EO or NE in the supernatant (mg/mL).

#### 2.6.3. Determination of pH

The pH of the NP suspensions was measured using a benchtop pH meter (Model HI 2221; Hanna Instruments, Smithfield, RI, USA), calibrated with standard buffer solutions of pH 4, 7, and 10. The NPs were dispersed in ultrapure water to ensure sample homogeneity. The pH meter probe was immersed in the NP solution, and readings were taken after the pH value stabilized to minimize interference or fluctuations.

#### 2.6.4. Scanning Electron Microscopy

The shape of the NPs obtained was analyzed using scanning electron microscopy (Zeiss EVO MA10 SEM, Oberkochen, Germany). The colloidal suspension (10 µL) was deposited in stubs, followed by 24 h air-drying. After total solvent evaporation, samples were coated with a 5 nm Au/Pd layer by sputtering. The microscopy conditions were set as follows: a working distance (WD) of 8.5 mm, magnification of 20,000×, an ion probe current (I Probe) of 20 pA, and examination at an accelerating voltage of 10 kV.

### 2.7. Antifungal Activity of Nanoparticles

#### 2.7.1. NP Contact Method

The antifungal activity of the NPs was determined using the direct contact method on in vitro agar. This method was similar to that described in the “Agar Contact Method” Section 2.4.1, with the free compounds being replaced by the NP-EO and NP-NE.

#### 2.7.2. Spore Germination Assay

To evaluate the effect of NPs on spore germination, the method described by Zhao et al. [34] with modifications was followed. Spores of *B. cinerea* or *Rhizopus* sp. were suspended in 1 mL of Tween 80 (0.05%) using a Drigalski loop and collected in a sterile microtube. The spore concentration was 1 × 10^5^ CFU/mL, quantified using a Neubauer chamber. The spores were incubated in Potato-Dextrose broth containing NP-EO or NP-NE at concentrations that showed the best antifungal activity against the mycelium (10, 20, and 25 mg/mL) and incubated for 72 h at 25 °C. At least 100 spores were counted to calculate the germination rate.

### 2.8. Storage Stability Analysis

To evaluate the storage stability of NP-EO or NP-NE under temperature stress conditions, the NPs were prepared as described in the “NP Preparation” section. The prepared NPs were then stored at different temperatures (4 °C, 25 °C, and 37 °C) at pH 7.0. Samples were collected on days 0, 3, 5, 7, 10, 13, and 15 for size analysis using a Nanotrac NPA150 (Microtrac Inc., York, PA, USA) DLS-based device.

### 2.9. Hemolysis Assay

Hemolytic activity was evaluated essentially as described previously [33]. The NPs were mixed at a 1:1 (*v*/*v*) ratio in heparinized sheep blood (4%, *v*/*v* in PBS, pH 7.4). The aliquots were incubated (60 min at 37 °C) and then centrifuged at 3000× *g* for 10 min. One mL of the supernatant was taken for reading on a spectrophotometer at 540 nm. Triton X-100 (0.1%, *v*/*v*) and PBS were used as positive and negative controls, respectively [35]. Hemolytic activity was determined with Equation (3):

(3) $$Hemolytic\;activity\;\left(\%\right)=\frac{\left(AS-AN\right)}{\left(AP-AN\right)}\times 100$$

where AS: sample reading, AN: negative control, AP: positive control.

### 2.10. Statistical Analysis

Experimental data were expressed in the form of mean ± standard deviation. The analysis software used was GraphPad Prism (Version 6.0, GraphPad Software Inc., Boston, MA, USA). For comparisons between two groups, the *t*-test was applied. For comparisons between three or more groups, one-way analysis of variance (ANOVA) was performed, followed by Tukey’s multiple comparisons post hoc test to identify significant differences. In all analyses, *p*-values lower than 0.05 were considered statistically significant.

## 3. Results

### 3.1. Essential Oil Characterization

The chromatographic profile of the *B. dracunculifolia* EO is detailed in Table S1. Nerolidol (NE) was the major compound in the EO, with a relative area of 12.80%. Other major compounds present in the EO (>3.00% in area) included: biciclogermacrene (12.40%), β-pinene (10.91%), spathulenol (10.07%), β-caryophyllene (8.29%), trans-α-bergamotene (6.73%), α-pinene (3.56%), and δ-cadinene (3.29%). Minor compounds in the EO had relative areas ranging from 0.23% to 2.99%. With 28 identified compounds, the total relative area of the identified peaks amounted to 97.61%. Sesquiterpenes constituted a major portion of the total compounds (60.71%), followed by monoterpenes (21.43%), sesquiterpenoids (10.71%), monoterpenoids (3.57%), and sesquiterpenoid esters (3.57%).

### 3.2. Antifungal Activity of EO and NE

#### 3.2.1. Agar Contact Method

**Figure 3:**
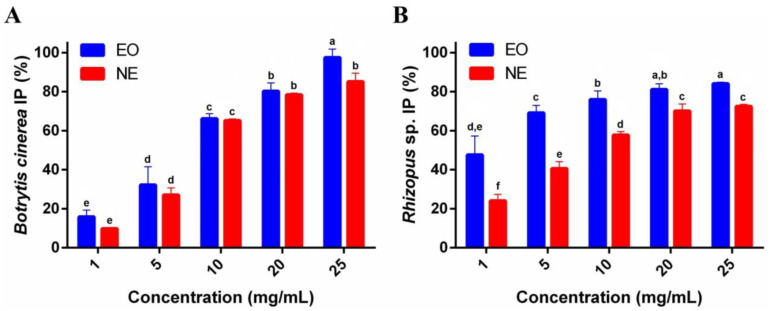
Antifungal activity of free EO and NE against *B. cinerea* (**A**) and *Rhizopus* sp. (**B**) after 7 days of growth at concentrations ranging from 1 to 25 mg/mL. Results are the means ± standard deviations of three independent experiments. Different letters over the bars indicate significant differences (*p* < 0.05).

The antifungal activity of free EO and NE against *B. cinerea* after 7 days of growth is presented in Figure 3A. For EO, the highest inhibition percentages were 80.3 ± 4.2% and 97.5 ± 4.3% at concentrations of 20 and 25 mg/mL, respectively. Similarly, NE at the same concentrations achieved IP values of 78.4 ± 0.8% and 85.2 ± 4.3%.

The antifungal activity of free EO and NE against *Rhizopus* sp. after 7 days of growth is shown in Figure 3B. For all concentrations tested, EO showed higher IP values as compared with NE. The IP increased from 69.2 ± 3.7% to 84.1 ± 0.6% when the EO concentration varied from 5 to 25 mg/mL. In the same concentration range, the maximum inhibition of NE was 72.5 ± 0.9% at 25 mg/mL.

These results demonstrate substantial antifungal activity for both EO and NE, with higher antifungal activity at higher concentrations, particularly against *B. cinerea* (*p* < 0.05). In contrast, NE was effective against *Rhizopus* sp. but showed lower inhibition even at the highest concentration tested. These findings suggest that EO is more effective than NE, particularly against *Rhizopus* sp., and that both exhibit concentration-dependent antifungal activity.

#### 3.2.2. Exposure to Volatiles

**Figure 4:**
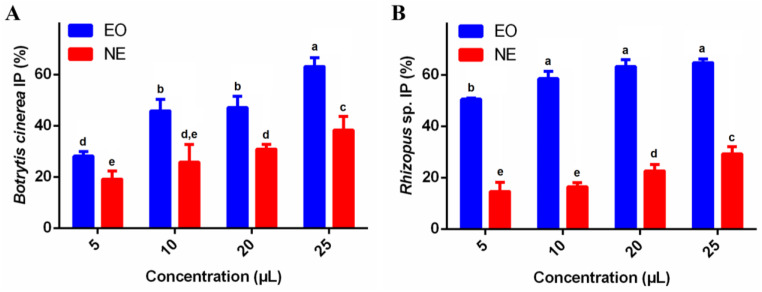
Antifungal activity of volatile fractions of EO and NE against *B. cinerea* (**A**) and *Rhizopus* sp. (**B**) after 7 days of incubation in different concentrations. Results are the means ± standard deviations of three independent experiments. Different letters over the bars indicate significant differences (*p* < 0.05).

The results of *B. cinerea* and *Rhizopus* sp. exposure to EO and NE volatiles revealed variability in efficacy across different concentrations (Figure 4). For *B. cinerea*, EO demonstrated increased inhibitory activity with higher concentrations, ranging from 28.2 ± 1.8% to 63.2 ± 3.4% at concentrations ranging from 5 to 25 µL. In contrast, NE exhibited lower efficacy, with a maximum inhibition of 38.4 ± 5.2% at 25 µL. Regarding *Rhizopus* sp., EO also showed superior efficacy, with an inhibition percentage of 50.5 ± 0.5% at the lowest concentration (5 µL), reaching 64.8 ± 1.4% at 25 µL. While less effective, NE showed an increase in inhibition, ranging from 14.6 ± 3.6% to 29.3 ± 2.8% at the same concentrations. These results suggest that EO volatiles exhibit more pronounced antifungal activity compared to NE volatiles, particularly against *Rhizopus* sp., with a more effective response as the concentration of volatiles increases.

### 3.3. Characterization of Nanoparticles

**Figure 5:**
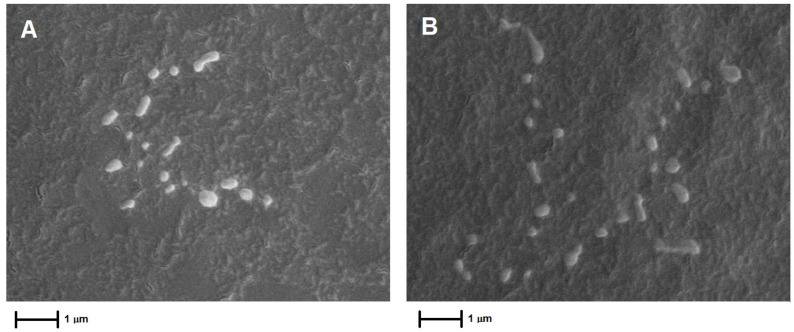
Scanning electron microscopy (SEM) images of Pluronic^®^ F-127 nanoparticles loaded with EO (**A**) and NE (**B**) at 20,000× magnification. Bar = 1 μm.

The NPs were characterized in terms of particle size, PDI, zeta potential (ζ), EE, and pH (Table 1). NP-EO exhibited an average diameter similar to the control nanoparticle (NP-B), while NP-NE showed a larger average size. Despite these observed differences in size, they were not statistically significant. The polydispersity of NP-NE was similar to that observed for the control NPs (NP-B), but the PDI values were significantly different from NP-EO. Moreover, the histograms of size distribution (Figure S1) showed that the formulations had a monomodal distribution profile and 90% (*D*_0.9_) of the NPs presented diameters smaller than 200 nm. NP-NE exhibited a more negative zeta potential compared to NP-EO and NP-B (*p* < 0.05), indicating higher surface charge, although all formulations maintained relatively low zeta potential values. The encapsulation efficiency was significantly higher for NP-EO compared to NP-NE. In terms of pH, NP-EO and NP-NE presented higher pH values than NP-B. Finally, SEM analysis revealed that the NPs were spherical in shape, consistent with the particle size estimates obtained by DLS (Figure 5).

### 3.4. Antifungal Activity of NP-EO and NP-NE

#### 3.4.1. Agar Contact Method

**Figure 6:**
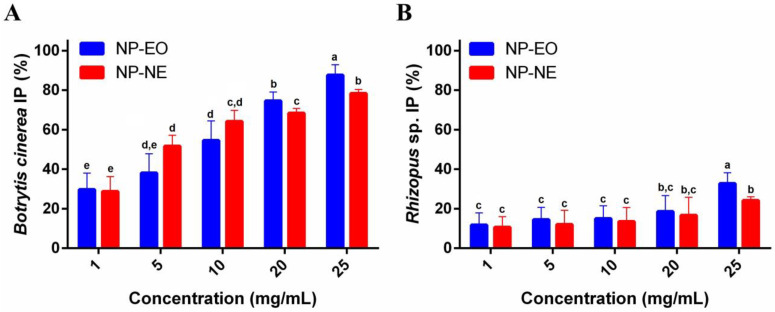
Antifungal activity of nanoparticles (NP-EO and NP-NE) against *B. cinerea* (**A**) and *Rhizopus* sp. (**B**) after 7 days of growth at concentrations ranging from 1 to 25 mg/mL. Results are the means ± standard deviations of three independent experiments. Different letters over the bars indicate significant differences (*p* < 0.05).

The NPs were assessed for their antifungal activity against *B. cinerea*, with concentrations ranging from 1 to 25 mg/mL, as shown in Figure 6A. NP-EO demonstrated antifungal activity, with inhibition increasing significantly from 29.7 ± 8.3% at 1 mg/mL to 87.6 ± 5.3% at 25 mg/mL, showing a clear dose-dependent effect. NP-NE also exhibited considerable antifungal activity, with inhibition ranging from 28.7 ± 7.5% to 78.4 ± 2.0%. Overall, both NP-EO and NP-NE formulations showed enhanced antifungal activity as the concentration increased, with clear differences between the lowest and highest concentrations for each formulation.

The NPs were also evaluated for their antifungal activity against *Rhizopus* sp., with concentrations varying from 1 to 25 mg/mL, as detailed in Figure 6B. NP-EO exhibited antifungal activity in a concentration-dependent profile, with inhibition percentages ranging from 11.8 ± 6.1% to 32.7 ± 5.4%. Similarly, NP-NE showed increased fungal inhibition, ranging from 10.7 ± 5.3% to 24.2 ± 1.8%. Generally, NP-EO formulations demonstrated similar antifungal potential compared to NP-NE at all tested concentrations.

#### 3.4.2. Spore Germination

The results of spore germination tests for *B. cinerea* and *Rhizopus* sp. exposed to different concentrations of NP-EO and NP-NE indicate a reduction in the spore germination rate with increasing concentrations of both treatments (Table 2).

For *B. cinerea*, the untreated control samples (0 mg/mL) showed a spore germination percentage of around 66%. When NP-EO was added at concentrations between 10 and 25 mg/mL, a progressive reduction in spore germination was observed, with inhibition ranging from moderate to nearly complete. A similar pattern was seen for NP-NE across the same concentration range. For *Rhizopus* sp., spore germination in the untreated samples was between 73 and 78%. The addition of NP-EO also resulted in significant spore germination inhibition, although the effect was less pronounced than for *B. cinerea*. NP-NE showed comparable inhibitory effects, with a dose-dependent response similar to NP-EO. Overall, both nanoparticle formulations demonstrated efficacy in inhibiting spore germination.

### 3.5. Storage Stability of Nanoparticles

**Figure 7:**
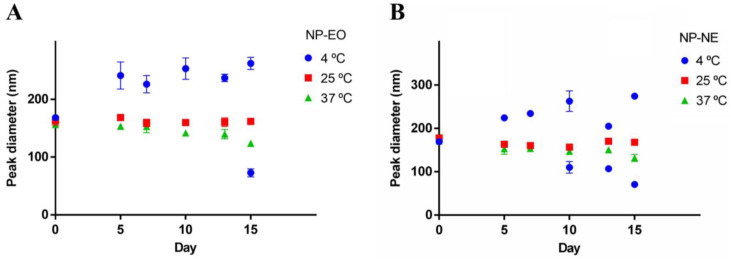
Hydrodynamic size variations of NP-EO (**A**) and NP-NE (**B**) over 15 days at different temperatures measured by DLS. Results are the means ± standard deviations of three independent experiments.

The changes in the hydrodynamic particle size of the NP-EO and NP-NE measured by DLS over 15 days at different temperatures are shown in Figure 7. At 25 °C, the particle sizes remained stable throughout the storage period, indicating good stability at room temperature. In contrast, NPs stored at 4 °C exhibited different size profiles compared to those stored at 25 °C, particularly after the fifth day. For NP-NE stored at 4 °C, a second particle population with an average diameter of approximately 110.10 ± 13.29 nm was detected on the 10th day, decreasing to 107.0 ± 0.00 nm by the 13th day and further to 70.90 ± 6.93 nm by the 15th day (Figure 7B).

Similarly, a second particle population was also observed in NP-EO stored at 4 °C, emerging on the 15th day with a size of 72.85 ± 6.86 nm. Unlike the particles stored at 25 °C, where stability was preserved, those stored at 4 °C demonstrated a trend towards forming smaller populations over time. Additionally, at 37 °C, NP-EO sizes decreased starting from the 10th day, suggesting possible degradation processes. Although most NP-NE maintained their original size at 37 °C, a trend for size reduction was also noted. These observations indicate that while NPs are stable at 25 °C, both lower (4 °C) and higher (37 °C) temperatures induce the modification of particle size and affect the overall stability of the formulations during storage.

### 3.6. Hemolytic Activity

**Figure 8:**
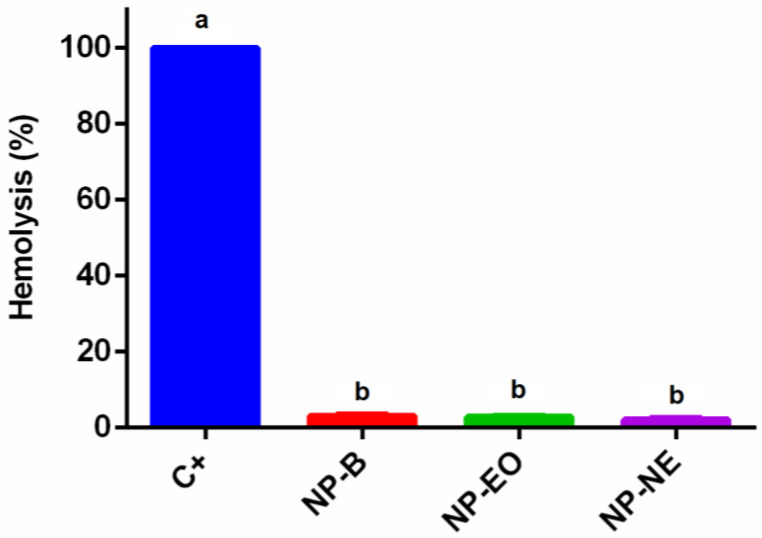
Hemolytic activity of NP-B, NP-EO, and NP-NE compared to the positive control (C+). Results are the means ± standard deviations of three independent experiments. Different letters over the bars indicate significant differences (*p* < 0.05).

The hemolytic activity of NP-B, NP-EO, and NP-NE compared to the positive control (C+) is presented in Figure 8. NP-B exhibited low hemolysis levels, with a value of 3.19 ± 0.86%. Similarly, NP-EO and NP-NE also showed low hemolysis, with values of 2.87 ± 0.83% and 2.18 ± 1.08%, respectively. These results suggest that while NP-B demonstrates low hemolysis, the inclusion of EO and NE in the NPs did not result in an increase in hemolytic activity (*p* < 0.05).

## 4. Discussion

The EO of *B. dracunculifolia* has nerolidol as its major component, which is consistent with previous reports [15,33]. The presence of β-caryophyllene and β-pinene is also notable, given their association with antifungal activities [36]. This suggests that the antifungal activity observed could be partially attributed to the synergistic action of these major components, especially NE, which has shown potent antifungal effects against filamentous fungi, such as various *Aspergillus* and *Fusarium* species [36,37].

The results of antifungal activity indicated significant inhibition of *B. cinerea* and *Rhizopus* sp. by both EO and NE. EO was particularly effective against *B. cinerea*, achieving over 50% inhibition at intermediate concentrations and over 95% at the highest concentration. NE also showed good activity, although less effective, particularly at higher concentrations. These results highlight the pronounced antifungal activity of EO compared to NE, similar to that described for other EOs [29,38,39]. The results of volatile exposure further supported the superior antifungal activity of EO over NE, showing a concentration-dependent response for both fungi. This may be due to the synergistic effects of the volatile components in EO, as terpenes and monoterpenes can work together to enhance fungal inhibition [40]. Previous studies have demonstrated the synergistic effects of compounds such as linalool and caryophyllene in EO from *Michelia alba* [41]. Thus, the combined effects of EO components are greater than the sum of individual effects, as seen with the compound NE. Additionally, volatiles showed significantly lower inhibitory activity than the direct contact method. This difference arises because free compounds in the solid medium ensure a more consistent concentration, while the varying volatilities of individual EO compounds cause them to diffuse at different rates in a non-saturated environment, resulting in higher emission rates that can reduce efficacy [42].

Both EO and NE were effectively incorporated in Pluronic NPs. The use of ultrasound was important for achieving nanometer-sized particles during NP preparation. The ultrasonic energy broke larger droplets, promoting the formation of more stable and smaller NPs [43]. Although the nature of the encapsulated compounds may influence particle size due to specific interactions with the polymer matrix, both EO and NE caused no significant effects on this parameter, as NP-EO and NP-NE exhibited similar particle diameters to that observed for the control (NP-B). Comparable particle sizes ranging from 100 to 200 nm have been reported for different NP formulations encapsulating EOs [25,33].

Furthermore, PDI values suggest that the NPs prepared in this study showed high uniformity. Polydispersity is an indicative of the homogeneity of particle size distribution, and PDI values around 0.2–0.3 have been associated with narrow size distribution [22].

The zeta potential results from the arrangement of the materials used in the formulation, and a relatively high value is important to maintain the physicochemical stability of the colloidal suspension since large repulsive forces tend to hinder aggregation of adjacent NPs [44]. The incorporation of EO or NE improved the zeta potential of the bare NPs, as NP-B had a zeta potential close to zero, indicating low colloidal stability. NP-NE exhibited a more negative zeta potential as compared to NP-EO, suggesting higher colloidal stability for NP-NE due to greater electrostatic repulsion. EO droplets present negative zeta potential values [45], and therefore a negative zeta potential is generally expected for NPs with an oily core. However, despite the higher negative charge of NP-NE, which could enhance penetration into fungal cells, NP-EO showed better antifungal activity. This discrepancy may be attributed to the specific chemical composition of the EO, which contains active compounds with strong antimicrobial properties, as reported in previous studies [40,41]. While zeta potential influences colloidal stability, other factors, such as the bioactive properties of the encapsulated compounds, play a crucial role in determining antifungal efficacy.

The EE varied considerably, with NP-EO achieving about 80%, significantly higher than NP-NE at 51%. The complex composition of EO, rich in monoterpenes, appears to facilitate better cohesion and packing of nonpolar chains within the NP vesicles, enhancing stability and retention. Monoterpenes located in the polar regions of membranes influence surface curvature and promote higher encapsulation efficiency [46]. NE, being a single compound, may have limited interactions with Pluronic^®^ F-127, resulting in higher leakage during encapsulation and lower EE. The EE of NE is consistent with those observed for other individual EO components, such as timol and carvacrol, showing 46.3% and 50.9%, respectively [47].

The NPs (NP-EO and NP-NE) demonstrated improvements in antifungal efficacy as compared to non-encapsulated compounds, indicating potential for food preservation against fungal contaminants [48]. Encapsulation allows for controlled release of active compounds, resulting in prolonged effects, which is crucial for food storage and shelf life [22,49]. NP-EO, for instance, showed up to 87% inhibition of *B. cinerea*, comparable to or exceeding non-encapsulated EO. Moreover, NP-EO effectiveness at relatively low concentrations, such as 1 mg/mL, indicates its potential for the development of active packaging and preservatives [48]. This suggests that encapsulation not only preserved but may have enhanced the antifungal efficacy of EO, offering a robust solution for food protection against pathogenic fungi [22,49,50]. NP-NE also showed good efficacy, with up to 78% inhibition. This difference may be due to the higher volatility of NE, which is partially mitigated by encapsulation, allowing for sustained and protected release of the active compound [37,51].

There is limited literature on the inhibition of *Rhizopus* species by *B. dracunculifolia* EO and NE. However, the results of this study indicate that for *Rhizopus* sp., NPs were less effective as compared to *B. cinerea*. NP-EO achieved a maximum inhibition of 32%, while NP-NE reached 24%, suggesting that *Rhizopus* sp. is more resistant to treatments. This point is relevant for the preservation of foods where this fungus might be present, such as strawberries, peaches, tomatoes, and sweet potatoes [19,52,53]. In this case, further optimization of the NP formulation would be necessary to increase the efficiency against this fungus.

The effectiveness of NP-EO and NP-NE in inhibiting spore germination reflects their ability to halt fungal spread and infection, particularly in fresh foods such as tomatoes and strawberries [19,39]. The NPs showed pronounced inhibition of spore germination, especially at higher concentrations, suggesting their potential effectiveness in preventing fungal contamination from the early stages of sporulation. These findings reinforce the potential of NPs as active additives in food preservation, offering an innovative approach for extending shelf life and ensuring food quality [54,55].

Overall, the NPs demonstrated promising effectiveness, with the potential to enhance the delivery of bioactive compounds such as EO and NE. The ability of NPs to protect active compounds from degradation and volatilization may explain their increased or comparable efficacy to non-encapsulated compounds [48,50]. NP-EO, in particular, performed better than NP-NE, especially against *B. cinerea*, likely due to its chemical composition and higher EE, resulting in greater retention of bioactive compounds and enhanced antifungal efficacy in both contact and spore germination assays [43].

The stability analysis indicated that NP-EO and NP-NE formulations maintained good stability at 25 °C, with no significant changes in hydrodynamic sizes over 15 days. This demonstrates that at room temperature, the NPs can maintain their physicochemical characteristics, which is favorable for storage and transportation under normal conditions. This makes them suitable for application in active food packaging that does not require refrigeration [56,57]. However, storage at 4 °C led to the formation of a secondary population of particles, suggesting some instability at lower temperatures. This effect is potentially due to decreased hydrophobicity of the poly(oxpropylene) at lower temperatures, which might have caused the loss of oil emulsification and micelle disintegration [22]. At elevated temperatures (37 °C), NP-EO showed a significant decrease in size, possibly indicating degradation processes from prolonged exposure, facilitating drug release [58]. In contrast, NP-NE showed a constant size during incubation, suggesting greater resistance to thermal degradation [26]. These data are important for evaluating the stability of formulations under various conditions, which is crucial for ensuring their long-term efficacy.

The hemolytic activity results indicate the NPs have a safe potential for use in food applications and packaging. All NP formulations showed values below 3%, indicating minimal damage to erythrocytes. This safety profile is crucial, as it suggests that NPs containing EO and NE do not increase cytotoxicity, which is an important factor for their potential future use in food preservation, active packaging, and antimicrobial films where consumer safety is a priority [57,59]. However, it is essential to conduct further research on the potential side effects of nanoparticle migration from packaging to food, as well as to carry out preclinical and clinical studies to validate the safety of these nanoparticles.

## 5. Conclusions

NP-EO demonstrated superior antifungal activity compared to NP-NE. NP-EO significantly inhibited the growth of *B. cinerea* and moderately inhibited *Rhizopus* sp. in contact assays, while also showing substantial spore germination inhibition. This enhanced performance is attributed to the complex composition of *B. dracunculifolia* EO, which includes bioactive compounds such as nerolidol, β-caryophyllene, and α-pinene, resulting in robust antifungal efficacy and high EE. In contrast, NP-NE had a lower EE and exhibited reduced antifungal effectiveness, likely due to limited interaction between NE and Pluronic^®^ F-127, affecting encapsulation and retention efficiency. The low hemolytic activity of the NPs indicates their safety for food and packaging applications. These findings highlight the efficacy and applicability of NP-EO encapsulated with *B. dracunculifolia* EO in extending food shelf life and ensuring product quality. The controlled and prolonged release of active compounds, coupled with their antifungal activity and safety, suggests that these NPs could be an innovative and promising approach for food preservation and active packaging development after approval by regulatory agencies.

## Figures and Tables

**Table 1:** Physicochemical characteristics of nanoparticle suspensions containing *B. dracunculifolia* essential oil (NP-EO), Nerolidol (NP-NE), and the control (NP-B) *.

Nanoparticle	Mean Size (nm)	PDI	ζ (mV)	pH	EE (%)
NP-EO	114.90 ± 0.17 ^a^	0.303 ± 0.04 ^a^	−4.01 ± 1.30 ^a^	6.64 ± 0.08 ^a^	80.09 ± 1.23 ^a^
NP-NE	123.07 ± 10.9 ^a^	0.234 ± 0.08 ^b^	−16.35 ± 0.04 ^b^	5.95 ± 0.38 ^a^	51.07 ± 0.78 ^b^
NP-B	113.23 ± 0.91 ^a^	0.258 ± 0.03 ^b^	−0.165 ± 0.18 ^c^	5.47 ± 0.04 ^b^	-

* Different letters in columns indicate significant differences (*p* < 0.05). Abbreviations: EE, encapsulation efficiency; PDI, polydispersity index; ζ, zeta potential.

**Table 2:** Spore germination percentage of *B. cinerea* and *Rhizopus* sp. treated with different concentrations of NP-EO and NP-NE.

	Spore Germination (%) *	
Nanoparticle (mg/mL)	*Botrytis cinerea*	*Rhizopus* sp.
Control	66.33 ± 6.43 ^Aa^	73.33 ± 5.51 ^Aa^
NP-EO (10)	45.00 ± 5.30 ^Ab^	47.33 ± 3.79 ^Ab^
NP-EO (20)	15.67 ± 3.22 ^Ac^	25.00 ± 3.61 ^Bc^
NP-EO (25)	2.33 ± 0.58 ^Ad^	6.67 ± 1.16 ^Bd^
Control	65.00 ± 6.56 ^Aa^	78.00 ± 1.00 ^Ba^
NP-NE (10)	42.33 ± 2.52 ^Ab^	50.67 ± 2.08 ^Bb^
NP-NE (20)	16.00 ± 5.00 ^Ac^	24.33 ± 5.85 ^Ac^
NP-NE (25)	3.33 ± 1.16 ^Ad^	9.00 ± 1.73 ^Bd^

* For each treatment, means followed by different uppercase letters in the rows and different lowercase letters in the columns indicate significant differences (*p* < 0.05).

## Data Availability

The original contributions presented in the study are included in the article/Supplementary Material, further inquiries can be directed to the corresponding author.

## Supplementary Materials

The following supporting information can be downloaded at: https://www.mdpi.com/article/10.3390/foods13213403/s1, Figure S1: Histograms of particle size distribution obtained by laser dynamic light scattering for Pluronic^®^ F-127 nanoparticles loaded with *Baccharis dracunculifolia* EO (A) and nerolidol (B); Table S1: Chemical composition of *Baccharis dracunculifolia* EO.
