# Supplementary material for: Inhibition of Food Spoilage Fungi, Botrytis cinerea and Rhizopus sp., by Nanoparticles Loaded with Baccharis dracunculifolia Essential Oil and Nerolidol
Source: Foods. 2024 Oct 25;13(21):3403. doi: 10.3390/foods13213403 (PMC11544775; doi:10.3390/foods13213403)
Supplement: Supplementary file 1 [file foods-13-03403-s001.zip › foods-3266391-supplementary.pdf]

foods

Supplementary material

Inhibition of food spoilage fungi by nanoparticles loaded with *Baccharis dracunculifolia* essential oil and nerolidol

Aldrey Nathália Ribeiro Corrêa, Naiara Jacinta Clerici, Natália Oliveira de Paula, and Adriano Brandelli

Supplementary Table S1

Table S1. Chemical composition of *Baccharis dracunculifolia* EO.

Compound	Relative area (%)	Similarity (%)	RT
α-Pinene	3.56	97.98	6.40
Sabinene	0.23	95.55	7.55
β-Pinene	10.41	97.34	7.67
β-Myrcene	0.91	97.02	8.05
Limonene	8.42	98.57	9.27
cis-β-Ocimene	0.37	94.28	9.88
α-Terpineol	0.21	95.36	14.73
β-Bourbonene	0.23	95.32	21.25
β-Elemene	0.85	95.35	21.45
α-Gurjunene	0.23	96.52	22.04
β-Caryophyllene	8.29	98.99	22.35
Aromadendrene	0.79	96.54	22.95
Humulene	1.38	97.13	23.41
Alloaromadendrene	2.11	95.69	23.63
γ-Muurolene	2.02	96.96	24.10
trans-α-Bergamotene	6.73	95.15	24.26
Bicyclogermacrene	12.40	97.12	24.74
β-Bisabolene	0.57	95.95	25.04
γ-Cadinene	0.57	95.71	25.23
δ-Cadinene	3.29	96.81	25.50
Nerolidol	12.80	95.53	26.66
Palustrol	0.46	97.37	26.84
Spathulenol	10.07	95.39	27.13
Globulol	2.99	97.98	27.30
Viridiflorol	4.91	95.67	27.55
β-Guaiene	1.00	95.22	27.86
τ-Cadinol	0.87	96.22	28.92
τ-Cadinol Acetate	0.94	97.62	29.28

Supplementary Figure S1

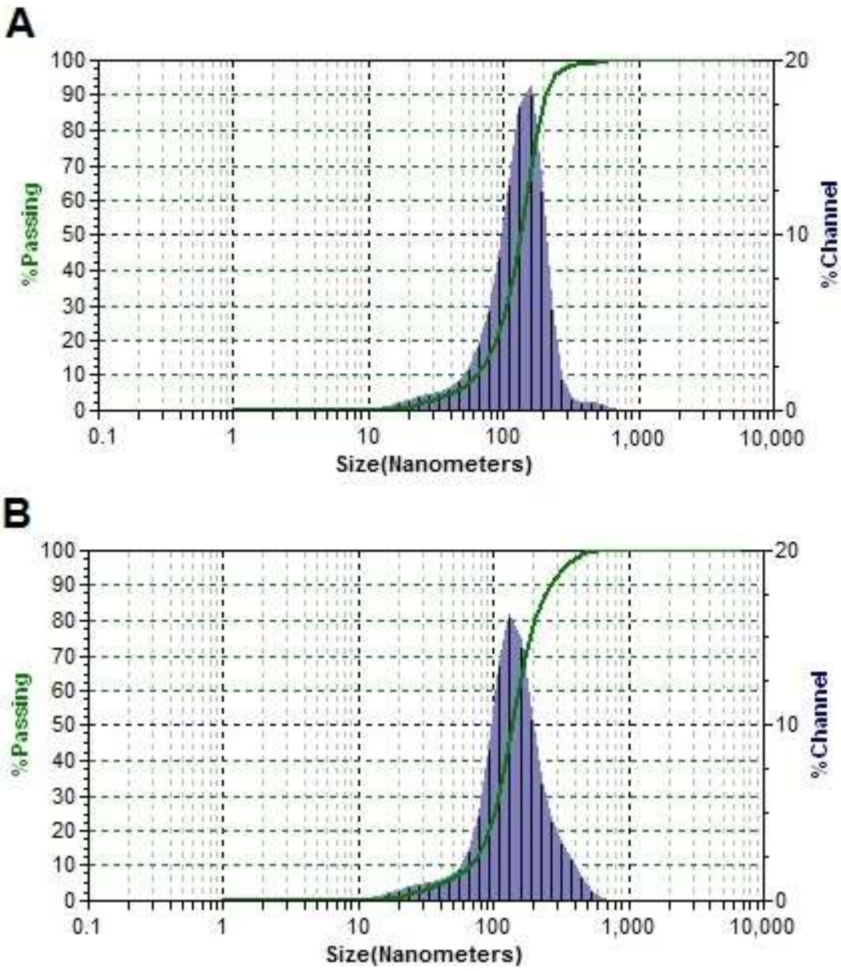

Figure S1. Histograms of particle size distribution obtained by laser dynamic light scattering for Pluronic® F-127 nanoparticles loaded with *Baccharis dracunculifolia* EO (A) and nerolidol (B).
